# Analysis of embryo morphokinetics, multinucleation and cleavage anomalies using continuous time-lapse monitoring in blastocyst transfer cycles

**DOI:** 10.1186/1477-7827-12-54

**Published:** 2014-06-20

**Authors:** Nina Desai, Stephanie Ploskonka, Linnea R Goodman, Cynthia Austin, Jeffrey Goldberg, Tommaso Falcone

**Affiliations:** 1 Department of Reproductive Endocrinology and Infertility, Cleveland Clinic, Beachwood, Ohio, USA

**Keywords:** Blastocyst, Time-lapse, Morphokinetic parameters, Implantation, Embryo development, Pregnancy, Embryoscope, Cleavage

## Abstract

**Background:**

Time-lapse imaging combined with embryo morphokinetics may offer a non-invasive means for improving embryo selection. Data from clinics worldwide are necessary to compare and ultimately develop embryo classifications models using kinetic data. The primary objective of this study was to determine if there were kinetic differences between embryos with limited potential and those more often associated with in vitro blastocyst formation and/or implantation. We also wanted to compare putative kinetic markers for embryo selection as proposed by other laboratories to what we were observing in our own laboratory setting.

**Methods:**

Kinetic data and cycle outcomes were retrospectively analyzed in patients age 39 and younger with 7 or more zygotes cultured in the Embryoscope. Timing of specific events from the point of insemination were determined using time-lapse (TL) imaging. The following kinetic markers were assessed: time to syngamy (tPNf), t2, time to two cells (c), 3c (t3), 4c ( t4), 5c (t5), 8c (t8), morula (tMor), start of blastulation (tSB); tBL, blastocyst (tBL); expanded blastocyst (tEBL). Durations of the second (cc2) and third (cc3) cell cycles, the t5-t2 interval as well as time to complete synchronous divisions s1, s2 and s3 were calculated. Incidence and impact on development of nuclear and cleavage anomalies were also assessed.

**Results:**

A total of 648 embryos transferred on day 5 were analyzed. The clinical pregnancy and implantation rate were 72% and 50%, respectively. Morphokinetic data showed that tPNf, t2,t4, t8, s1, s2,s3 and cc2 were significantly different in embryos forming blastocysts (ET or frozen) versus those with limited potential either failing to blastulate or else forming poor quality blastocysts ,ultimately discarded. Comparison of embryo kinetics in cycles with all embryos implanting (KID+) versus no implantation (KID-) suggested that markers of embryo competence to implant may be different from ability to form a blastocyst. The incidence of multinucleation and reverse cleavage amongst the embryos observed was 25% and 7%, respectively. Over 40% of embryos exhibiting these characteristics did however form blastocysts meeting our criteria for freezing.

**Conclusions:**

These data provide us with a platform with which to potentially enhance embryo selection for transfer.

## Background

In vitro fertilization (IVF) success rates have increased since 1978 and the birth of the first IVF baby. The implantation rate per embryo transferred in patients under 35 in 2011 was still however only 36%, according to the SART (Society of Assisted Reproduction) national IVF registry. In spite of ASRM guidelines to reduce the number of embryos transferred, many centers still transfer 2-3 embryos despite the increased risk of multiple pregnancy, with its associated neonatal and maternal complications, in order to maximize the chance for pregnancy (SART.org).

The most critical step during IVF is embryo selection for transfer. For the last two decades, the conventional method of embryo selection for transfer has been based on critical assessment of morphologic parameters during embryonic development. Currently, these morphological assessments are limited to once a day at set time points, since repeated removal of embryos from the incubator environment for observation may result in undesired temperature and pH shifts in the embryo culture dish.

Embryo development is a dynamic event and static observations of embryonic growth can therefore be limiting in their ability to discern differences between embryos at similar cell stages. The introduction of time-lapse imaging and monitoring systems in the clinical IVF laboratory has allowed more detailed observations on embryo developmental kinetics. Numerous data suggest that the precise timing of specific events such as pronuclear formation, syngamy, early cleavage events, cell cycle intervals, synchronicity of cell division and initiation of blastulation are indicators of an embryo’s developmental potential [1-7]. The ability to continuously monitor an embryo’s progression towards these benchmarks may therefore aid in selecting the best embryos for uterine transfer. These published data suggest that morphokinetic observations can yield valuable information to aid the selection of embryos for transfer. The question still to be answered is whether the reported observations can be universally applied to all IVF clinics, without regard to culture methodology. Inability to effectively apply a published embryo selection model to another setting was demonstrated by Best el al [8]. Impact of single versus sequential culture media systems on time-lapse data also needs to be considered. Contradictory conclusions were reached in two recent studies looking at the relationship between culture media and embryo kinetics [9,10]. To validate the clinical use of time lapse technology for embryo selection each laboratory needs to first characterize optimal growth patterns for human embryos within their own in vitro culture system.

The specific aims of this study were: (1) to present our initial clinical outcome data with continuous time-lapse imaging in the Embryoscope, (2) to determine if there are kinetic differences between embryos with limited developmental potential and those forming blastocysts of a quality suitable for transfer or freezing , (3) to determine if there are differences in kinetics between implanting and non-implanting blastocysts and (4) to compare these observations to embryo selection markers proposed by other laboratories.

## Methods

### Study design

This was a retrospective study of prospectively acquired data of time-lapse imaging of human embryos during in vitro growth. This research was carried out at the Cleveland Clinic Fertility Center between April and November 2012. The study was approved by our Institutional Review Board and was carried out following ethical guidelines set forth by our institution. Patients with 10 or more mature oocytes and at least 7 fertilized oocytes were offered the opportunity to participate in this investigation. The study was restricted to this subset of patients to only include those who had enough embryos that selection parameters could potentially aid in embryo selection for transfer. The exclusion criteria for this study were maternal age over 39 and cases involving surgically retrieved sperm. A total of 81 patients were enrolled. The number of patients included in the study was determined by the total number of patients recruited during six months of data collection. The data set analyzed included 648 embryos, derived from 60 patients, cultured until the blastocyst stage (day 5-6). The remaining 21 patients had a day 3 transfer and their morphokinetic data was excluded. Time-lapse images of human embryo development were critically assessed by one of three observers, the lab director and two senior embryologists. At the outset of the study all three observers viewed videos as a group and established annotation guidelines. Consistency of grading and annotation were thereafter reviewed by the lab director and discussed at weekly lab meetings.

### Ovarian stimulation and oocyte retrieval

Women were treated with either a GnRH-agonist (gonadotropin releasing hormone) or a GnRH-antagonist to suppress ovulation until follicle maturity was attained. Ovarian stimulation was initiated using daily injections of follicle stimulating hormone (FSH) with starting doses based on serum anti-Mullerian hormone levels, antral follicle counts and previous responses to ovarian stimulation. The subsequent doses were adjusted according to follicle growth and serum estradiol levels. Final follicular maturation was triggered with human chorionic gonadotrophin (hCG) when at least two lead follicles measured 18 mm in mean diameter. Oocytes were collected 36 hours later by transvaginal needle aspiration of follicles under ultrasound guidance. Oocytes were placed in a 35 mm dish containing 25 μl drops of Global fertilization medium (LifeGlobal; Guilford, CT) supplemented with 10% human serum albumin (Sage/Cooper-Surgical, Trumball, CT) under an oil overlay. Dishes were incubated at 37˚C with 6% CO_2_ and 21% atmospheric oxygen in a Forma incubator.

### Oocyte retrieval, ICSI and embryo culture

Oocytes were denuded of cumulus cells 2-3 hours after the retrieval using hyaluronidase (Sage). Intracytoplasmic sperm injection (ICSI) was then performed on mature oocytes. Injected oocytes were moved to pre-equilibrated 25 μl drops of Global medium with 10% Synthetic Protein Supplement (SPS; Cooper Surgical; Trumbull, CT) under an oil overlay and cultured overnight at 37˚C with 6% CO_2_ and 21% atmospheric oxygen.

An EmbryoSlide™ (Fertiitech, Inc., Rockland MA) was prepared on the day of egg retrieval for zygote culture after the fertilization check. The EmbryoSlide™ containing 12 individual wells was prepared by filling each well with 25 μl of Global medium with 10% SPS and overlaying with 1.2 ml of washed oil. The slide was equilibrated overnight.

Oocytes were examined 16-18 hours after insemination for signs of fertilization and the presence of two pronuclei. Zygotes were moved to the wells of the pre-equilibrated EmbryoSlide™. Care was taken to remove any bubbles using a finely drawn glass micropipette before placing the zygotes in the wells. Slides containing zygotes were placed in the Embryoscope chamber immediately following fertilization check and cultured for up to 6 days. Embryos were cultured at 37°C at 6% CO_2_ with 5.5% oxygen. Medium was refreshed on day 3 by performing a half-change with freshly prepared, pre-equilibrated culture medium. An Eppendorf pipette was used to withdraw 12 μl of fluid from each well and this was replaced with fresh medium, taking care not to disturb the embryo. The image acquisition software collected images of each embryo as it developed during the six day culture interval. The time-lapse video created from these images was used to monitor cell cleavage anomalies as well as the timing of specific cell cycle events.

### Time-lapse imaging system

The Embryoscope, recently FDA cleared in the U.S. for clinical embryology, was used for time lapse imaging of the embryos. It is a tri-gas incubation chamber equipped with a built in microscope (Hoffman Modulation contrast objective) and a high definition camera (1280 × 1024 pixels, 3 pixels μm), allowing continuous monitoring of embryonic growth. The chamber design and camera software is capable of imaging up to 72 embryos (6 patient slides with 12 embryos). The image acquisition system was set to capture high contrast 200× images from 5-7 focal planes for each embryo, every 15 minutes.

### Embryo assessment

Observations were made daily in the morning using the Embryoscope viewer. The time of ICSI was designated as time zero (t0). All timings were thereafter expressed as hours post-insemination (hpi) and zygotes were first placed in the Embryoscope at 17-18 hpi. For day 2 embryos, observations were made 42-44 hpi, day 3 at 66-68 hpi, day 4 at 90-92 hpi, day 5 at 114-116 hpi and day 6 at 138-140 hpi. Embryos were first graded at these set time points without viewing the accumulated time-lapse images. Information was recorded in the patient’s laboratory chart.

Cleavage stage embryos were assessed for cell stage, percent fragmentation, multinucleation and blastomere symmetry [11,12]. Increase in cell: cell adherence between blastomeres resulting in the merging of cells or “compaction” was also monitored. Embryos were scored as either compacting or else morula if over 90% of cells were merged [11,12]. Blastocyst grade was assigned based on the day of blastocyst formation, blastocyst maturity, inner cell mass development, and trophectoderm organization using a previously described scoring system [13]. Blastocoel volume and expansion were used to classify blastocysts as: A = early blastocyst, cavity just starting to form; B = early blastocyst, cavity less than half the volume of embryo; C = expanded with cavity greater than half embryo volume; D = fully expanded; and E = hatching. The inner cell mass was graded as: 0- absent or not yet visible; 1- sparse, few cells, loosely organized; and 2- well defined, discrete cell mass. Trophectoderm (TE) of the blastocyst was assessed based on cell number and organization: 1- low cell number, stretched appearance; 2- well organized cohesive cell layer; and 3- extremely high cell number and well organized. Presence of degenerative cells or dark grainy regions within the blastocyst were considered negative traits. Blastocysts having low TE cell number and degenerative cells in either TE or ICM were designated as “poor quality” and not considered suitable for transfer or freezing.

Blastocysts were selected for transfer based on conventional grading criteria without the use of kinetic data. Good quality blastocysts arising from embryos displaying an optimal growth pattern (i.e. 4 cell on day 2, 8 cell day 3, morula day 4, with fragmentation <25%) were given preference. Selection was based on blastocyst grade in the morning at 114-116 hpi even though transfers were generally scheduled for late afternoon. In patients presenting with only a single suitable quality blastocyst (n = 8), morula were transferred along with the blastocyst. Morula observed with beginnings of fluid were often early blastocysts by the time of transfer. Supernumerary blastocysts Grade B-E with an inner cell mass and a trophectoderm of good cell number and quality were cryopreserved. Time-lapse videos were reviewed in detail once conventional grading had been completed.

### Evaluation of time-lapse imaging and kinetics

Time lapse microscopy (TLM) allowed the visualization of nuclear and cytoplasmic events during the entire in vitro culture interval. Using the Embryo Viewer software, video records were annotated for the timing of specific developmental stages, cleavage anomalies and blastomere nuclearity from the point of ICSI. The following early kinetic markers were assessed: time to pronuclear fading or syngamy (tPNf), time to 2 cells (c) (t2), 3c (t3), 4c (t4), 5c (t5), 8c (t8) and 9c + or partially compacting (t9). We also looked at late kinetic parameters coinciding with genomic activation, specifically, morula (tMor), start of blastulation (tSB), blastocyst (tBL), and expanded blastocyst (tEBL). Embryos were labeled as morula when greater than 90% of the embryo was compacted and individual blastomeres could no longer be identified. Start of blastulation was defined as the earliest frame in which fluid could be distinctly seen amongst blastomeres in the morula stage embryo. Embryos were annotated as blastocysts when a crescent shaped region with fluid could be visualized. Expansion of the blastocyst in diameter was the point identified as tEB. Durations of the second cycle (cc2; t3-t2), third cell cycle (cc3; t5-t3) as well as intervals between 4 and 5 cells (t4int; t5-t4) and t5-t2 were also calculated. The final kinetic parameters looked at were the time to complete synchronous divisions s1 (t2-tPNf), s2 (t4-t3) and s3 (t8-t5).

Two cleavage anomalies specifically monitored were: reverse cleavage (RCLV), where a blastomere was re-absorbed after cleavage and direct cleavage (DC), where a single blastomere divided directly from 1 to 3 cells in less than 5 hours, as described by Rubio et al [14]. Presence of multinucleation (MU) within blastomeres was also recorded. The exact timing of cleavage anomalies and appearance of multinucleated blastomeres through the entire growth interval were carefully noted. In cases of multinucleation, we recorded number of blastomeres affected and number of nuclei per blastomere (MU2, binucleated; MU3+, 3 or more nuclei).

### Embryo transfer and clinical outcomes

Embryo transfer was performed on day 5 under trans abdominal ultrasound guidance using a Wallace Sure-View catheter. Patients used either intravaginal progesterone (Prometrium capsules; 200 mg/bid) or intramuscular injections of progesterone in oil (100mg) for luteal support starting the day of oocyte retrieval. Supernumerary blastocysts of good morphology, displaying an inner cell mass and adequate trophectodermal cells were cryopreserved.

Serum beta-hCG levels were measured 15 days after transfer. A clinical pregnancy was defined as visualization of an intrauterine gestational sac with fetal heart activity on ultrasound 5 weeks after the embryo transfer. The implantation rate (IR) was calculated by dividing the number of embryos with cardiac activity by the number of embryos transferred.

### Data analysis

For the purpose of data analysis cultured embryos were divided in to three groups: (A) Blastocysts transferred (BL-T) (B) Blastocysts frozen (BL-F) and (C) Poor quality blastocysts and non-blastulating embryos ultimately discarded (PQ-D). Kinetic data was compared between the three groups. We also looked specifically at transfers with known implantation data (KID). Timing of developmental endpoints were compared between transfers where all embryos implanted (KID+) and those in which all embryos failed to implant (KID-). The mean timing of cell division and cell cycle intervals were compared using the ANOVA parametric test and the Student t-test. The chi square test was used for analysis and comparison of proportions. P-values of <0.05 were considered statistically significant.

## Results

Patient demographics and cycle characteristics are shown in Table 1. The mean patient age was 33.5 ± 4.0 years. While only patients with 10 or more mature oocytes and at least 7 fertilized oocytes were included to ensure there would be an adequate number of embryos to review for selection, both good and poor prognosis patients were included. This is evident by the fact that 22% of patients recruited had at least two prior IVF failures (Table 1). The 648 zygotes generated were cultured to day 5 or 6 and monitored for blastocyst formation. Table 2 shows our first clinical outcome data with this new time-lapse imaging chamber. All patients having a transfer had at least one blastocyst transferred with a mean of 1.9 ± 0.8 embryos being transferred. The clinical pregnancy rate for day 5 transfers was 72% (41/57). The implantation rate per embryo transferred was 50% (61/121). Three patients displayed symptoms of ovarian hyperstimulation and were unable to have a transfer. Their blastocysts were therefore cryopreserved.

**Table 1:** Patient demographics and cycle characteristics

**Patients**	**60**
**Age**	33.5 ± 4.0 year
**Diagnosis**	
*Male Factor*	15 (25%)
*Unexplained*	13 (22%)
*Mixed Male and Female*	13 (22%)
*Tubal Factor*	7 (11%)
*Ovulatory Dysfunction*	5 (8%)
*Diminished Ovarian*	4 (7%)
*Reserve*	3 (5%)
*Endometriosis*	
**Cycle Type**	
*Agonist*	42 (70%)
*Antagonist*	18 (30%)
**Cycle Number**	
*1,2*	47 (78%)
*3‒6*	13 (22%)
**Mature Oocytes**	13.0 ± 4.3
**Oocytes fertilized by ICSI**	9.7 ± 3.3

**Table 2:** Clinical outcome data after culture in the Embryoscope

**Patients**	**60**^**a**^
Transfers	57^b^
Embryos cultured (TL)	11.0 ± 2.8
Embryos transferred	1.9 ± 0.8
Positive pregnancy test	81% (46/57)
Clinical pregnancy	72% (41/57)
Implantation rate	50% (61/121)
Live birth rate	68% (39/57)
Infants delivered	59
* Singleton deliveries*	21
* Twin deliveries*	19
Total TL embryos examined	648
* Blastocysts Transferred*	105
* Blastocysts Frozen*	335
* PQ Blastocysts/Non-blastulating*	208
Blastocysts transferred/frozen	68%

^a^Additional 21 patients enrolled in TL study having Day 3 transfer CPR 65%, IR 35%.

^b^Three cycles with no transfer due to hyper stimulation/all blastocysts frozen.

PQ = poor quality.

The 648 cultured zygotes were divided in to three groups (A-C) according to quality and final disposition as described earlier. A total of 335 blastocysts (BL-F) were frozen and 208 embryos (PQ-D) were discarded. Group A designated as BL-T, represents each patient’s best quality blastocysts selected for transfer. Amongst the 105 blastocysts transferred 69% were expanded blastocysts (Grade C or D), 31% were early blastocysts (Grade A or B). The timings of cleavage and specific early developmental endpoints amongst embryos from the three groups are shown in Table 3. Timing of syngamy (tPNf), s1 and early cell division to the 2,4 and 8 cell stage (t2,t4,t8) were significantly different in blastocysts (transferred or frozen) versus PQ-D blastocysts/non-blastulating embryos ultimately discarded (Group C). The time to reach synchrony after initiation of the second and third cleavage (s2 and s3) was also significantly shorter in Groups A and B versus C. Paradoxically, Group C embryos exhibited a much shorter “resting phase” between the 2-3 cell stage (cc2) and between the 4- 5 cell stage(t4int) before continuing to divide. Blastocysts selected for transfer were observed as ≥9 cells or partially compacted by 73.5 hpi (CI95%, 71.4-75.5) and morula at 93.9 hpi (CI95%, 91.9-95.9). Embryos in Group C were distinctly slower to reach the morula stage.

**Table 3:** Embryos kinetics during development to day 5

	**Blasts transferred (BL-T) Mean ± SD**	**95% CI**	**Blasts frozen (BL-F) Mean ± SD**	**95% CI**	**PQ BL/Non blasts (PQ-D) Mean ± SD**	**95% CI**	**p**
*tPNf*	24.8 ± 2.6	24.3- 25.3	25.2 ± 3.0	24.9 - 25.6	26.8 ± 8.3	26.6 - 28.0	0.001
*t2*	27.2 ± 3.6	26.5 - 27.8	27.7 ± 4.0	27.1-28.2	30.0 ± 8.8	28.8- 31.2	<0.0001
*t3*	37.6 ± 5.5	36.5-38.7	38.0 ± 5.8	37.4 - 38.6	38 ± 11.8	36.4- 39.6	ns
*t4*	40.0 ± 5.4	39.0 - 41.1	40.9 ± 6.1	40.3- 41.6	43.4 ± 1 4.3	41.4 - 45.4	0.003
*t5*	52.0 ± 6.3	50.8 - 53.2	52.4 ± 9.0	51.5 - 53.4	50.2 ± 15.3	48.0 - 52.4	ns
*t8*	62.1 ± 9.8	60.2- 64.0	63.8 ± 11.7	62.6- 65.1	71.1 ± 21.2	67.8 - 74.3	<0.0001
*t9+*	73.5 ± 10.3	71.4-75.6	74.7 ± 13.3	73.2-76.3	83.7 ± 24.5	79.7-87.8	<0.0001
*tMor*	93.9 ± 9.8	91.9 - 95.9	98.2 ± 12.8	96.8 - 99.7	110.7 ± 15.3	106.7 - 114.7	<0.0001
*tSB*	100.2 ± 7.4	99.0– 101.5	105.5 ± 10.3	104.5 – 106.5	113.1 ± 13.1	110.2 – 116.1	<0.001
*tBL*	105.2 ± 6.3	103.8 - 106.6	111.1 ± 10.5	109.9 - 112.3	121.4 ± 9.1	116-126.0	<0.0001
*tEBL*	110.0 ± 5.6	108.6 - 111.4	118.9 ± 12.1	117.3 - 120.4	133.1 ± 9.0	127.1-139.2	<0.0001
*cc2 (t3-t2)*	10.4 ± 4.5	9.6- 11.3	10.2 ± 4.7	9.7- 10.8	8.6 ± 10.7	7.1 - 10.1	0.02
*cc3 (t5-t3)*	14.4 ± 5.4	13.4-15.4	14.4 ± 7.3	13.6-15.1	14.0 ± 13.1	12.1-15.8	ns
*S1 (t2-tPNf)*	2.4 ± 1.7	2.0 – 2.7	2.6 ± 1.9	2.4 -2.8	3.2 ± 3.9	2.6 – 3.7	0.03
*s2 (t4-t3)*	2.4 ± 4.7	1.5 - 3.4	2.9 ± 5.0	2.4 -3.5	5.3 ± 8.9	4.1- 6.6	<0.0001
*s3 (t8-t5)*	10.2 ± 8.7	8.4- 11.9	11.5 ± 10.2	10.4 -12.6	21.8 ± 19.1	18.9 - 24.8	<0.0001
*t4int (t5-t4)*	12.0 ± 5.7	10.9-13.1	11.5 ± 6.5	10.8-12.2	8.7 ± 10.2	7.2-10.1	<0.0001
*t5-t2*	24.9 ± 5.8	23.8-26.0	24.6 ± 7.8	23.8-25.4	21.9 ± 16.2	19.7-24.2	<0.01

Embryos being cultured for blastocyst transfer cycles (n = 648) were divided into three groups: (A) BL-T, blastocysts transferred, (B) BL-F, blastocysts.

frozen and (C) PQ-D, Poor quality blastocysts/embryos failing to blastulate. Mean timings in BL-T and BL-F blastocysts(Group A, B) shown and compared to Group C. P value <0.05 was considered to be significant.

**Figure 1:**
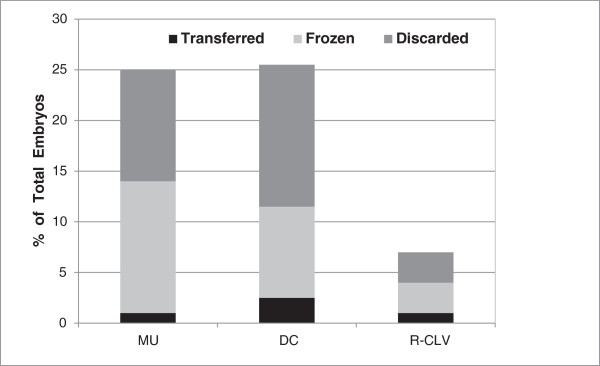
**Incidence of multinucleation and cleavage anomalies.** Graph depicts incidence of multinucleation and cleavage anomalies amongst the study group of 648 embryos. Y axis value represents the percent of embryos observed to have each of these anomalies. The diagram also shows how these features affected subsequent embryo development and the ultimate disposition of the embryo. Embryos developing to good quality blastocysts were either frozen or transferred.

To better understand the impact of different embryo kinetic parameters on ability to implant we looked at transfer cycles with “known implantation data” (KID). We had 38 KID patients in which all transferred blastocysts either implanted (KID+) or failed to implant (KID-). Table 4 contrasts the kinetics amongst the 68 transferred embryos with known implantation outcomes. Early morphokinetic parameters significantly associated with implanting vs. non-implanting blastocysts were t-TPNf, t2, t3, t5 ,t8 , s1 and t5-t2. There were no significant differences in cc2, cc3, s2, or s3 or the interval between 4 and 5-cell. Observations made possible through the time-lapse videos and of specific interest to us were the presence of multinucleation, reverse cleavage and direct cleavage in the cohort of embryos studied. The prevalence of these dysmorphisms and how they impacted subsequent embryo development and blastulation is depicted in Figure 1. The final disposition of embryos with each trait is also shown. With time lapse imaging we appear to be detecting a far higher percentage of multinucleation (25%) than with conventional observation on day 2 at 42 hpi (<5%). Amongst the multinucleated embryos 16% were binucleated and 9% had blastomeres with 3 or more nuclei. The incidence of reverse cleavage and direct cleavage (DC) was 7%, and 26%, respectively. These dysmorphisms often presented together in embryos of poor quality. We noted that amongst embryos exhibiting direct cleavage and/or reverse cleavage, at least one fourth were also multinucleated. Interestingly, approximately 40% of embryos displaying reverse cleavage, 56% of binucleated (MU2+) embryos and 48% of MU3+ embryos continued on to make blastocysts that met our criteria for freezing. The implantation potential of these embryos is yet unclear as they were under represented in the cohort of embryos selected for fresh transfer.

**Table 4:** Kinetics in implanting (KID+) versus non-implanting (KID-) embryos selected for day 5 transfer

	**KID + implanting mean ± SD**	**KID - non-implanting mean ± SD**	**p**
*tPNf*	24.1 ± 2.5	26.2 ± 2.7	0.001
*t2*	26.8 ± 3.8	28.5 ± 4.2	0.02
*t3*	36.5 ± 4.7	40.1 ± 6.8	0.004
*t4*	39.3 ± 3.7	42.6 ± 7.5	ns
*t5*	51.0 ± 4.8	54.0 ± 6.2	0.02
*t8*	59.6 ± 9.1	63.9 ± 9.8	0.02
*t9+*	72.3 ± 11.7	75.2 ± 10.3	ns
*tMor*	90.5 ± 8.9	95.6 ± 10.6	ns
*tSB*	98.1 ± 7.0	99.3 ± 8.6	ns
*tBL*	102.9 ± 6.8	105.7 ± 6.2	ns
*tEBL*	109.9 ± 6.4	109.8 ± 5.1	ns
*cc2 (t3-t2)*	9.7 ± 4.0	11.6 ± 5.5	ns
*cc3 (t5-t3)*	14.5 ± 4.1	13.9 ± 6.5	ns
*s1 (t2-tPNf)*	3.2 ± 3.9	2.3 ± 2.3	0.04
*s2 (t4-t3)*	2.8 ± 4.4	2.6 ± 6.7	ns
*s3 (t8-t5)*	8.7 ± 7.5	10.3 ± 8.2	ns
*t4int (t5-t4)*	11.7 ± 3.6	11.7 ± 5.6	ns
*t5-t2*	24.2 ± 4.1	25.9 ± 6.0	0.03

Comparison of kinetics in KID + and KID– transfers. Implanting and non-implanting blastocysts showed significant differences in early but not late kinetic parameters. P values <0.05 were considered to be significant.

## Discussion

IVF outcome data from national registries like SART give evidence to the wide variation in clinical outcomes amongst clinics. Certainly differences in laboratory protocols and expertise factor in to these data and the ultimate implantation potential of embryos being generated. It is therefore of paramount importance that laboratories collect time-lapse image information and first establish baseline kinetics for embryo development within their own clinical and laboratory setting.

This study represents our first experience with the use of the Embryoscope and time-lapse imaging for embryo culture in our laboratory. The controlled environment provided within the time lapse-chamber proved to be beneficial for cultivation of human embryos resulting in high day 5 pregnancy outcomes. Our initial findings suggest that early morphokinetic parameters do in fact differ between embryos making good quality useable blastocysts as compared to those embryos failing to blastulate or showing poor blastocyst morphology. Furthermore even amongst good quality transferred blastocysts, we were able to detect differences amongst implanting versus non-implanting blastocysts using time-lapse data. This latter observation may be especially valuable as our laboratory moves towards elective single embryo transfer (SET).

To date, there have only been a few studies looking at the relationship between early kinetics, blastulation and implantation potential [2,4,5,7,15-17]. Using thawed pronuclear zygotes donated for research, Wong et al [7] developed an algorithm for selecting embryos likely to reach blastocyst based on the duration of the first cytokinesis (P1), the time between the first and second mitosis (P2, also termed cc2), and the time between second and third mitoses to reach synchrony (P3, also termed s2). Conaghan et al., using a computer automated time-lapse system, reported that the combination of two kinetic endpoints P2 and P3 with conventional day 3 morphology grading could improve the embryologist’s ability to select embryos most likely to develop into useable blastocysts.

Embryo kinetics for predicting blastocyst formation as opposed to implantation may potentially differ. Kirkegaard et al. reported that duration of the first cytokinesis, duration of the 3 cell stage and direct cleavage to 3 cell were useful parameters in predicting blastocyst formation [5]. At the same time these parameters could not identify differences between implanting and non-implanting embryos. Time interval before initiation of the third cleavage event (cc3) has also been suggested to be useful in selecting blastocysts likely to implant [1]. Meseguer and colleagues created a tiered system for embryo classification using kinetic parameters to predict implantation (after exclusion of embryos deemed non-viable) [6]. They used time to 5 cell (t5) as the primary criteria, followed by time to synchrony from 3c to 4c (s2) and duration of the second cell cycle (cc2) as their third criteria. Interestingly this group went on to apply these same criteria to predict blastocyst formation, and found that the best indicator for discriminating between viable embryos (those developing to blastocyst) and non-viable embryos was t5 and late cell division events [15]. However, it is important to note that the morphokinetic data analyzed were from donor oocyte cycles and cleavage timings may differ in embryos derived from an infertile patient population [18]. Herrero et al, from the same laboratory group more recently published a large data set describing the kinetic pattern of development in embryos from both oocyte donors as well as infertile patients having day 3 or day 5 transfers [17]. Their extensive analysis showed that t5 and t8 were more indicative of continuing viability to the blastocyst stage than early kinetic parameters like t2, t3, t4 which they suggest may be only predictive of short term development.

In the present study with 648 embryos from an infertile patient population, we did not find cc3 or t5 to be helpful in discriminating between embryos making blastocysts versus those of limited viability either failing to blastulate or developing in to poor quality, unusable blastocysts. At the same time, It should also be pointed out that the t5 values (mean 52.0, 95% CI 50.8-53.2) in our transferred blastocysts were quite in line with those observed by Herrero et al in their grouping of blastocysts with optimal morphology [17]. In the current analysis, other early kinetic parameters, namely tPNf, t2, t4, t4int, t8, s1,s2, cc2 and t5-t2 were found to be distinctly different between non-viable embryos and high potential embryos capable of developing in to good quality blastocysts. Morphokinetic differences between viable and non-viable embryos were accentuated with time in culture. Kinetics of morulation (tMor), start of blastulation (tSB), blastocyst formation (tBL) and expansion (tEBL) were several hours faster than in embryos with limited growth potential.

Our initial KID data albeit limited are in agreement with other studies suggesting that kinetic markers associated with blastocyst formation may not necessarily be the same as those defining an embryo’s competence to implant. Clearly, ability to implant may be influenced by both patient-specific factors such as age, oocyte quality, endometrial receptivity, and sperm factor, as well as laboratory culture conditions influencing blastocyst quality and ploidy [9,10,18-21]. Implanting KID + embryos underwent syngamy (tPNf) sooner and the time to t2, t3, t5 and t8 was faster than in KID - embryos. We found that s1, the interval from syngamy to the first cleavage was shorter in non-implanting embryos (p = 0.04). Cell cycle intervals (cc2, cc3) and synchrony (s2, s3) were not however different between implanting and non-implanting blastocysts. KID + and KID- blastocysts did not differ in their time to morula, initiation of blastulation or blastocyst formation. One of the limitations of early kinetic markers in predicting implantation may be that they mostly reflect the maternal genome since embryonic genome activation and expression occur later between the 4-8 cell stage [22]. A more robust set of KID data is obviously needed to determine the clinical significance of these observations and whether early kinetic parameters can indeed be incorporated in to an algorithm for selection of blastocysts most likely to implant.

The combining of morphokinetics with preimplantation genetic screening (PGS) technology to develop a non-invasive model to identify euploid embryos has garnered a lot of interest. Hong et al observed a reduced prevalence of aneuploidy in blastocysts with early time to cavitation [23]. In a recent report, Basile et al propose an algorithm for increasing the probability of selecting chromosomally normal embryos using early kinetic data, specifically t5-t2 interval >20 hours and cc3 ranging between 11.7 and 18.2 hours [24]. The cc3 and t5-t2 intervals for good quality blastocysts as well as implanting blastocysts in the current work fell within their stated range. Campbell and colleagues looked at pre-implantation screening results after trophectoderm biopsy in combination with time-lapse data. They suggest that the start of blastulation (tSB) and time to reach a full blastocyst (tBL) may be used for blastocyst selection by classifying embryos according to risk for aneuploidy [25]. According to their model, embryos with tSB <96.2 hpi and tBL <122.9 hpi are at low risk for aneuploidy and at medium risk if tSB is greater than 96.2hpi with tBL <122.9 hpi. Interestingly, in their work euploid and aneuploid embryos did not differ in early kinetic parameters. In the present study, the kinetics for tSB and tBL in top tier blastocysts selected for transfer fell in the low to medium risk category. This was to be expected since we were selecting embryos that had reached the expanded blastocysts stage at our conventional observation point of 114 hpi on day 5 of culture. The association between aneuploidy and embryo growth is still however under debate. In a non time-lapse study, Kroener et al studying day 3 biopsied embryos found no relationship between timing of blastulation (day 5 vs. day 6) and aneuploidy [26]. Yet Alfarawat et al using trophectoderm biopsy for PGS of blastocysts found that poor trophectoderm development increased the probability of aneuploidy [27]. While the use of morphokinetics for assessing aneuploidy risk is an intriguing possibility, strong caution is also being urged as much more data is needed to substantiate the different proposed risk-models [28,29].

Culture media and specifically the use of single step versus sequential media may also contribute to the observed kinetic pattern within a cohort of embryos. Ciray et al observed that embryos cultured in a single step medium were advanced from the first mitosis thru to the 5-cell stage over sibling embryos cultured in sequential media but the cell cycle intervals (cc2, cc3) and s2 did not differ. Pregnancy and implantation rate were also not affected. In contrast Basile et al comparing single step medium (Global) to a sequential medium (Quinn’s Cleavage) observed no significant differences in timings or outcome parameters. The protocol in this initial TL study used Global as a single step medium with a medium refresh on Day 3. We have since shifted over to uninterrupted culture in Global medium from day 1 thru 5 with equally good clinical results [30]. In depth comparisons of implantation data from laboratories with differing culture protocols may shed more light on whether any observed differences in timings have clinical significance.

In addition to kinetic parameters, there are other events witnessed with time-lapse monitoring that may benefit embryo selection. The visualization of anomalous events such as direct cleavage and reverse cleavage previously not possible with conventional static microscopy may be useful as deselection criteria. Direct cleavage first studied by Rubio et al., was shown to be associated with lower implantation rates [14]. These investigators showed an implantation rate of only 1.2% in embryos dividing from 1 to 3c in less than 5 hours (t3-t2). Within our ET data set, blastocysts displaying DC were under represented (n = 16) and only partial implantation data was available as in many cases they were transferred with at least one non-DC embryo.

Increased detection of multinucleation with time-lapse morphology may be another tool for embryo deselection. Multinucleation may arise from karyokinesis without subsequent cytokinesis or alternatively from errors during mitosis in chromosome segregation and packaging, quite often resulting in chromosomally abnormal cells [31]. Data suggest that multinucleation impairs subsequent embryonic development and potential for implantation [32,33]. PGS studies have however also shown that not all multinucleated embryos are chromosomally abnormal [34]. Binucleated patterns of multinucleation may be less detrimental as a high percentage of such embryos appear to be euploid as compared to embryos presenting with 3 or more nuclei in a single blastomere [35]. From our data set it is clear that a large proportion of multinucleated embryos (MU 2 and MU3+) developed into blastocysts meeting our freezing criteria. Future transfer of frozen blastocysts derived from our multinucleated embryos may further elucidate the impact of this anomaly on embryo competence and implantation potential.

Laboratories acquiring new time lapse capabilities face the dilemma of how to move forward using the technology. This study’s sample size was limited to six months of data collection to allow for an expedient analysis of data to gain an understanding of the morphokinetic patterns within our own laboratory environment. A limitation of this study was its retrospective nature and restriction to a study population of patients with a larger number of embryos. A prospective randomized trial to see if conventional morphology combined with kinetics can help to “refine” selection of blastocysts on day 5 and increase implantation rates is currently underway (clinicaltrials.gov NCT02081859).

## Conclusions

The present work illustrates the importance of sequential observation of embryo developmental patterns and the wealth of additional information that can potentially be incorporated in to an embryo classification model.

## Competing interests

The authors declare that they have no competing interests.

## Authors’ contributions

ND designed the study, critically assessed data and drafted manuscript. SP analyzed data and performed statistical analysis. LG helped revise manuscript. JG, CA and TF reviewed manuscript. All authors read and approved the final manuscript.

## References

[B1] Chamayou S Patrizio P Storaci G Tomaselli V Alecci C Ragolia C Crescenzo C Guglielmino A The use of morphokinetic parameters to select all embryos with full capacity to implant J Assist Reprod Genet 2013 30 703 710 10.1007/s10815-013-9992-2 23585186 PMC3663978

[B2] Conaghan J Chen AA Willman SP Ivani K Chenette PE Boostanfar R Baker VL Adamson GD Abusief ME Gvakharia M Loewke KE Shen S Improving embryo selection using a computer-automated time-lapse image analysis test plus day 3 morphology: results from a prospective multicenter trial Fertil Steril 2013 100 412 419 e415 10.1016/j.fertnstert.2013.04.021 23721712

[B3] Cruz M Gadea B Garrido N Pedersen KS Martinez M Perez-Cano I Munoz M Meseguer M Embryo quality, blastocyst and ongoing pregnancy rates in oocyte donation patients whose embryos were monitored by time-lapse imaging J Assist Reprod Genet 2011 28 569 573 10.1007/s10815-011-9549-1 21394522 PMC3162049

[B4] Dal Canto M Coticchio G Mignini Renzini M De Ponti E Novara PV Brambillasca F Comi R Fadini R Cleavage kinetics analysis of human embryos predicts development to blastocyst and implantation Reprod Biomed Online 2012 25 474 480 10.1016/j.rbmo.2012.07.016 22995750

[B5] Kirkegaard K Kesmodel US Hindkjaer JJ Ingerslev HJ Time-lapse parameters as predictors of blastocyst development and pregnancy outcome in embryos from good prognosis patients: a prospective cohort study Hum Reprod 2013 28 2643 2651 10.1093/humrep/det300 23900207

[B6] Meseguer M Herrero J Tejera A Hilligsoe KM Ramsing NB Remohi J The use of morphokinetics as a predictor of embryo implantation Hum Reprod 2011 26 2658 2671 10.1093/humrep/der256 21828117

[B7] Wong CC Loewke KE Bossert NL Behr B De Jonge CJ Baer TM Reijo Pera RA Non-invasive imaging of human embryos before embryonic genome activation predicts development to the blastocyst stage Nat Biotechnol 2010 28 1115 1121 10.1038/nbt.1686 20890283

[B8] Best L Campbell A Duffy S Montgomerey S Fishel S Does one model fit all? Testing a published embryo selection algorithm on independent time-lapse data Hum Reprod 2013 28 suppl 1 i87 i90 10.1093/humrep/det190

[B9] Basile N Morbeck D Garcia-Velasco J Bronet F Meseguer M Type of culture media does not affect embryo kinetics: a time-lapse analysis of sibling oocytes Hum Reprod 2013 28 634 641 10.1093/humrep/des462 23315059

[B10] Ciray HN Aksoy T Goktas C Ozturk B Bahceci M Time-lapse evaluation of human embryo development in single versus sequential culture media–a sibling oocyte study J Assist Reprod Genet 2012 29 891 900 10.1007/s10815-012-9818-7 22714134 PMC3463674

[B11] Desai NN Goldstein J Rowland DY Goldfarb JM Morphological evaluation of human embryos and derivation of an embryo quality scoring system specific for day 3 embryos: a preliminary study Hum Reprod 2000 15 2190 2196 10.1093/humrep/15.10.2190 11006197

[B12] Machtinger R Racowsky C Morphological systems of human embryo assessment and clinical evidence Reprod Biomed Online 2013 26 210 221 10.1016/j.rbmo.2012.10.021 23352813

[B13] Desai N Kinzer D Loeb A Goldfarb J Use of Synthetic Serum Substitute and alpha-minimum essential medium for the extended culture of human embryos to the blastocyst stage Hum Reprod 1997 12 328 335 10.1093/humrep/12.2.328 9070721

[B14] Rubio I Kuhlmann R Agerholm I Kirk J Herrero J Escriba MJ Bellver J Meseguer M Limited implantation success of direct-cleaved human zygotes: a time-lapse study Fertil Steril 2012 98 1458 1463 10.1016/j.fertnstert.2012.07.1135 22925687

[B15] Cruz M Garrido N Herrero J Perez-Cano I Munoz M Meseguer M Timing of cell division in human cleavage-stage embryos is linked with blastocyst formation and quality Reprod Biomed Online 2012 25 371 381 10.1016/j.rbmo.2012.06.017 22877944

[B16] Hlinka D Kalatova B Uhrinova I Dolinska S Rutarova J Rezacova J Lazarovska S Dudas M Time-lapse cleavage rating predicts human embryo viability Physiol Res 2012 61 513 525 22881225 10.33549/physiolres.932287

[B17] Herrero J Tejera A Albert C Vidal C de Los Santos MJ Meseguer M A time to look back: analysis of morphokinetic characteristics of human embryo development Fertil Steril 2013 100 1602 1609 10.1016/j.fertnstert.2013.08.033 24083877

[B18] Bellver J Mifsud A Grau N Privitera L Meseguer M Similar morphokinetic patterns in embryos derived from obese and normoweight infertile women: a time-lapse study Hum Reprod 2013 28 794 800 10.1093/humrep/des438 23293223

[B19] Kirkegaard K Hindkjaer JJ Ingerslev HJ Effect of oxygen concentration on human embryo development evaluated by time-lapse monitoring Fertil Steril 2013 99 738 744 e734 10.1016/j.fertnstert.2012.11.028 23245683

[B20] Munoz M Cruz M Humaidan P Garrido N Perez-Cano I Meseguer M Dose of recombinant FSH and oestradiol concentration on day of HCG affect embryo development kinetics Reprod Biomed Online 2012 25 382 389 10.1016/j.rbmo.2012.06.016 22877941

[B21] Cruz M Garrido N Gadea B Munoz M Perez-Cano I Meseguer M Oocyte insemination techniques are related to alterations of embryo developmental timing in an oocyte donation model Reprod Biomed Online 2013 27 367 375 10.1016/j.rbmo.2013.06.017 23953584

[B22] Braude P Bolton V Moore S Human gene expression first occurs between the four- and eight-cell stages of preimplantation development Nature 1988 332 459 461 10.1038/332459a0 3352746

[B23] Hong KH Forman EJ Prodoehl A Upham KM Treff NR Scott JRT Early times to cavitation are associated with a reduced prevalence of aneuploidy in embryos cultured to the blastocyst stage: A prospective blinded morphokinetic study Fertil Steril 2013 100 811

[B24] Basile N Nogales Mdel C Bronet F Florensa M Riqueiros M Rodrigo L Garcia-Velasco J Meseguer M Increasing the probability of selecting chromosomally normal embryos by time-lapse morphokinetics analysis Fertil Steril 2014 101 699 704 10.1016/j.fertnstert.2013.12.005 24424365

[B25] Campbell A Fishel S Bowman N Duffy S Sedler M Hickman CF Modelling a risk classification of aneuploidy in human embryos using non-invasive morphokinetics Reprod Biomed Online 2013 26 477 485 10.1016/j.rbmo.2013.02.006 23518033

[B26] Kroener L Ambartsumyan G Briton-Jones C Dumesic D Surrey M Munne S Hill D The effect of timing of embryonic progression on chromosomal abnormality Fertil Steril 2012 98 876 880 10.1016/j.fertnstert.2012.06.014 22789142

[B27] Alfarawati S Fragouli E Colls P Stevens J Gutierrez-Mateo C Schoolcraft WB Katz-Jaffe MG Wells D The relationship between blastocyst morphology, chromosomal abnormality, and embryo gender Fertil Steril 2011 95 520 524 10.1016/j.fertnstert.2010.04.003 20537630

[B28] Ottolini C Rienzi L Capalbo A A cautionary note against embryo aneuploidy risk assessment using time-lapse imaging Reprod Biomed Online 2013 28 273 275 24433755 10.1016/j.rbmo.2013.10.015

[B29] Swain JE Could time-lapse embryo imaging reduce the need for biopsy and PGS? J Assist Reprod Genet 2013 30 1081 1090 10.1007/s10815-013-0048-4 23842747 PMC3790111

[B30] Rhambia P Desai N Global Medium is Effective as a Single One-Step Medium for Uninterrupted Culture to Blastocyst in the EmbryoScope: Preliminary Pregnancy and Clinical Outcome Data Fertil Steril Book Global Medium is Effective as a Single One-Step Medium for Uninterrupted Culture to Blastocyst in the EmbryoScope: Preliminary Pregnancy and Clinical Outcome Data 2014 101 e29

[B31] Pickering SJ Taylor A Johnson MH Braude PR An analysis of multinucleated blastomere formation in human embryos Hum Reprod 1995 10 1912 1922 8583010 10.1093/oxfordjournals.humrep.a136206

[B32] Ambroggio J Gindoff PR Dayal MB Khaldi R Peak D Frankfurter D Dubey AK Multinucleation of a sibling blastomere on day 2 suggests unsuitability for embryo transfer in IVF-preimplantation genetic screening cycles Fertil Steril 2011 96 856 859 10.1016/j.fertnstert.2011.07.1110 21851938

[B33] Van Royen E Mangelschots K Vercruyssen M De Neubourg D Valkenburg M Ryckaert G Gerris J Multinucleation in cleavage stage embryos Hum Reprod 2003 18 1062 1069 10.1093/humrep/deg201 12721185

[B34] Staessen C Van Steirteghem A The genetic constitution of multinuclear blastomeres and their derivative daughter blastomeres Hum Reprod 1998 13 1625 1631 10.1093/humrep/13.6.1625 9688403

[B35] Meriano J Clark C Cadesky K Laskin CA Binucleated and micronucleated blastomeres in embryos derived from human assisted reproduction cycles Reprod Biomed Online 2004 9 511 520 10.1016/S1472-6483(10)61635-5 15588469

